# Prediction of mortality in adult patients with severe acute lung failure receiving veno-venous extracorporeal membrane oxygenation: a prospective observational study

**DOI:** 10.1186/cc13824

**Published:** 2014-04-09

**Authors:** Tone Bull Enger, Alois Philipp, Vibeke Videm, Matthias Lubnow, Alexander Wahba, Marcus Fischer, Christof Schmid, Thomas Bein, Thomas Müller

**Affiliations:** 1 Department of Cardiothoracic Surgery, University Medical Center Regensburg, Franz-Josef-Strauss Allee 11, 93057 Regensburg, Germany; 2 Department of Internal Medicine II, University Medical Center Regensburg, Franz-Josef-Strauss Allee 11, 93057 Regensburg, Germany; 3 Department of Anaesthesiology, University Medical Center Regensburg, Franz-Josef-Strauss Allee 11, 93057 Regensburg, Germany; 4 Department of Laboratory Medicine, Children’s and Women’s Health, Faculty of Medicine, Norwegian University of Science and Technology, 7489 Trondheim, Norway; 5 Department of Circulation and Medical Imaging, Faculty of Medicine, Norwegian University of Science and Technology, 7489 Trondheim, Norway; 6 Department of Cardiothoracic Surgery, St. Olavs University Hospital, 7006 Trondheim, Norway; 7 Department of Immunology and Transfusion Medicine, St. Olavs University Hospital, 7006 Trondheim, Norway

## Abstract

**Introduction:**

Veno-venous extracorporeal membrane oxygenation (vvECMO) can be a life-saving therapy in patients with severe acute lung failure refractory to conventional therapy. Nevertheless, vvECMO is a procedure associated with high costs and resource utilization. The aim of this study was to assess published models for prediction of mortality following vvECMO and optimize an alternative model.

**Methods:**

Established mortality risk scores were validated to assess their usefulness in 304 adult patients undergoing vvECMO for refractory lung failure at the University Medical Center Regensburg from 2008 to 2013. A parsimonious prediction model was developed based on variables available before ECMO initiation using logistic regression modelling. We then assessed whether addition of variables available one day after ECMO implementation enhanced mortality prediction. Models were internally validated and calibrated by bootstrapping (400 runs). Predictive ability, goodness-of-fit and model discrimination were compared across the different models.

**Results:**

In the present study population, existing mortality prediction tools for vvECMO patients showed suboptimal performance. Evaluated before vvECMO initiation, a logistic prediction model comprising age, immunocompromised state, artificial minute ventilation, pre-ECMO serum lactate and hemoglobin concentrations showed best mortality prediction in our patients (area under curve, AUC: 0.75). Additional information about norepinephrine dosage, fraction of inspired oxygen, C-reactive protein and fibrinogen concentrations the first day following ECMO initiation further improved discrimination (AUC: 0.79, *P* = 0.03) and predictive ability (likelihood ratio test, *P* < 0.001). When classifying patients as lower (<40%) or higher (>80%) risk based on their predicted mortality, the pre-ECMO and day1-on-ECMO models had negative/positive predictive values of 76%/82% and 82%/81%, respectively.

**Conclusions:**

While pre-ECMO mortality prediction remains a challenge due to large patient heterogeneity, evaluation one day after ECMO initiation may improve the ability to separate lower- and higher-risk patients. Our findings support the clinical perception that chronic health condition, high comorbidity and reduced functional reserves are strongly related to survival during and following ECMO support. Renewed evaluation the first day after ECMO initiation may provide enhanced guidance for further handling of ECMO patients. Despite the usefulness of prediction models, thorough clinical evaluation should always represent the cornerstone in decision for ECMO.

## Introduction

For more than 40 years, veno-venous extracorporeal membrane oxygenation (vvECMO) has been used in critically ill patients with reversible acute lung failure (ALF). Following major improvements in ECMO technology and safety, the survival benefit shown in the CESAR trial [1], the usefulness of ECMO support during the influenza-A/H1N1-virus epidemic in 2009 to 2010 [2] and the increasing evidence that early ECMO may help to avoid substantial lung and consecutive organ injury [3-5], ECMO is now implemented more frequently and in a broader spectrum of patients.

Objective risk assessment is a valuable tool to aid the selection of therapeutic approach, to compare ECMO practice and success rates across different ECMO centers and to provide relatives with objective information about the patient's condition. Thus, there has been an increasing interest in identifying important prognostic factors for vvECMO. Published work includes two risk scores designed for pre-ECMO mortality prediction in patients with acute respiratory distress syndrome (ARDS), the ECMOnet score [6] and the PRESERVE score [7]. Presently, it is thought that multi-organ failure and sepsis play a more important role than the patient’s hemodynamic and respiratory state in the acute situation [6,8,9]. However, findings have been inconsistent. Limitations of previous studies include changes in ECMO technology and procedure over time, small study populations, large patient heterogeneity, limited registration of potentially important factors related to outcome, and application of statistical methods which may not take into account the random variability of patients, thus leading to results that have not been reproducible.

A large study population, application of up-to-date statistical methods designed to avoid overfitting and use of thorough internal and external validation are crucial steps in the development of reproducible ECMO risk scores to be integrated in clinical practice. Based on prospectively collected data from an institution where different forms of ECMO are now performed more than 100 times per year, we validated the existing risk scores and compared their predictive ability with the more general Sequential Organ Failure Assessment (SOFA) score frequently used in the intensive care unit (ICU). Furthermore, in accordance with the hypothesis that ECMO outcome is largely dependent on the patient’s extra-pulmonary status and general health condition, our aim was to examine data regarding clinical characteristics, respiratory and laboratory parameters, to develop two novel risk scores: one designed for pre-ECMO mortality prediction and one including parameters measured one day after ECMO implantation. We compared the different pre-ECMO mortality prediction models and investigated whether day 1 parameters could enhance predictive ability.

## Materials and methods

From 1 January 2008 to 15 July 2013, vvECMO was initiated in 304 consecutive adults with ALF refractory to conventional therapeutic modalities at the University Medical Center Regensburg (UKR), Germany. The general indication for vvECMO was either a ratio of partial arterial pressure of oxygen/fraction of inspired oxygen (PaO_2_/FiO_2_) <80 mmHg on a positive end-expiratory pressure of ≥16 cm H_2_O, or a refractory respiratory acidosis (pH <7.25), despite optimization of conservative therapy.

The ECMO circuit consisted of a centrifugal pump and a coated polymethylpentene oxygenator. In 99% of the patients, cannulation was performed percutaneously with Seldinger’s technique. In 75%, single-lumen cannulas were implemented with blood-flow from the femoral vein and to the jugular (80%), subclavian (15%) or contralateral femoral (4%) vein. Nineteen percent and six percent of the patients received a single double-lumen jugulo-jugular (Avalon Elite^®^, MAQUET Holding B.V. & Co KG, Rastatt, Germany) and femoro-femoral (Novaport^®^ twin, Novalung GmbH, Heilbronn, Germany) cannula, respectively. Further ECMO settings and patient management followed an institutional protocol as described previously [8].

Clinical data regarding pre-, intra- and post-ECMO characteristics and functions were prospectively registered in the UKR ECMO database. More detailed method descriptions are given in Additional file 1. All patients were followed up until in-hospital death (non-survivors) or hospital discharge (survivors). The study was approved by the local ethical committee of the UKR (reference number: 14-180-0051). The requirement for individual patient consent was waived as this study was based on anonymized data from routine care.

### Statistical analysis

Data are given as median (95% confidence intervals; CI) for continuous variables, and as frequency (percentage) for categorical variables. The Mann Whitney U-test and χ^2^ test were used to investigate differences between survivors and non-survivors for continuous and categorical variables, respectively. All tests were two-sided and *P*-values ≤0.05 were considered significant. Cases with missing values were excluded from the analyses. Statistical analyses were performed using SPSS (version 21.0, SPSS Inc., Chicago, IL, USA), Stata (version 12.1, StataCorp LP, College Station, TX, USA), SigmaPlot (version 12.0, Systat Software, Inc., San Jose, CA, USA), Minitab (version 15.1.30, Minitab Inc., State College, PA, USA) and the ‘rms’, ‘Hmisc’, ‘xtable’ and ‘party’ packages of *R* statistical software (version 3.0.1, R Foundation^a^).

#### ***Validation of external risk scores***

The external scores were tested in the present patient population as described in the original publications (see Additional file 2). SOFA was scored for each patient during data collection and stored in the database. ECMOnet and PRESERVE scores were calculated retrospectively. We found matching definitions for all variables except plateau pressure, which was not registered in our database due to the mode of mechanical ventilation used. Peak inspiratory pressure was used as a substitute. The discriminative abilities of the external risk scores were assessed by the area under the receiver-operating characteristics curves (AUC). Since poor fit may be explained by substantial differences in the study populations, predictors identified by the two scores were recalibrated using multivariate logistic regression analyses. The original estimates for the linear predictor variables, on which the additive ECMOnet and PRESERVE scores were based, were compared with the recalibrated coefficients and odds ratios, respectively. Clinical usefulness was evaluated using information from the original studies. For the ECMOnet score, we compared the sensitivity and specificity in our population using the authors’ proposed cut-off score for mortality of >4.5 as well as the estimated optimal cut-off in our population, calculated by the Youden index [10]. For the PRESERVE score, we compared mortality rates across groups of PRESERVE scores as described in the original study. We used a simple prognostic separation index (PSEP) to estimate the ability of the score to separate individuals into prognostic groups and compared the index across the cohorts to evaluate the reproducibility in new patients [11].

#### ***Model 1: Pre-ECMO mortality prediction***

Variable selection for prediction of in-hospital death combined literature review, clinical experience and hypotheses of potential influences on ECMO outcome as well as computer-based modelling (Additional file 3). A final parsimonious model was derived using logistic regression on the full dataset (n = 304), and further validated using bootstrap resampling with replacement (400 runs). Predicted probabilities of mortality were calculated based on bootstrapped coefficients (400 runs). Model performance was evaluated by plotting observed probabilities across deciles of predicted risk. Model discrimination was assessed with AUC, goodness-of-fit with a Hosmer-Lemeshow (HL) test, model overfitting by calculation of the shrinkage factor, and calibration by a calibration plot. The novel risk model was compared with the ECMOnet, PRESERVE and SOFA scores (Additional file 1).

#### ***Model 2: Mortality prediction on day 1***

For the second model, we added variables available one day after ECMO initiation to the final pre-ECMO model. The procedure of variable selection, validation and calibration of the final model was performed as described for Model 1. Models 1 and 2 were compared statistically by a likelihood ratio (LR) test. A simultaneous comparison of discrimination for all models (n = 241) was performed using the method for non-parametric correlated AUCs as proposed by DeLong *et al.*[12]. The novel day 1 risk model was also compared with the ECMOnet, PRESERVE and SOFA scores calculated with day 1 data (n = 236).

Clinical usefulness of the two novel models was evaluated by dividing the cohort into three groups based on predicted mortality risks: low (<40%), moderate (40% to 80%) and high (>80%) (Additional file 1). A simple PSEP was calculated to address separation between the lowest and highest risk groups. Negative and positive predictive values were calculated for the lowest and highest risk groups, respectively, as a measure of clinical validity.

## Results

### Study population

Overall survival until hospital discharge was 61.5%; 81 patients (27%) died during mechanical support and 36 patients (12%) died after weaning from ECMO. The best outcome was noted in trauma patients (74%), followed by primary lung failure (66%), extra-pulmonary lung failure and other causes (both 50%). Before ECMO initiation, non-survivors were longer hospitalized, had higher serum lactate concentrations, lower hemoglobin concentrations and more frequently showed signs of additional organ dysfunctions (pre-ECMO need for continuous veno-venous hemofiltration, higher bilirubin and liver enzyme concentrations). PaO_2_/FiO_2_, commonly used in the classification of ARDS severity, did not differ significantly between survivors and non-survivors. On the first day on ECMO, non-survivors showed less improvement of their respiratory failure, with higher minute ventilation, persisting low oxygenation and higher norepinephrine dosages. A comparison of survivors and non-survivors is given in Table 1 and Additional file 4.

**Table 1:** Comparison of survivors and non-survivors after ECMO

	**Non-survivors (n = 117)**	**Survival to discharge (n = 187)**	***P*-value**
**Baseline clinical characteristics**			
Age (years)	54 (50 to 57)	46 (43 to 48)	<0.001
Immunocompromised state^a^	37 (31.6%)	26 (13.9%)	<0.001
Sequential organ failure assessment score	13 (12 to 14)	11 (11 to 12)	0.001
Continuous veno-venous hemofiltration pre-ECMO	45 (38.5%)	36 (19.3%)	<0.001
Classification of acute lung injury^b^			0.022
- Group 1: Pulmonary	56 (47.9%)	107 (57.2%)	
- Group 2: Extra-pulmonary	36 (30.8%)	36 (19.3%)	
- Group 3: Trauma	10 (8.6%)	29 (15.5%)	
- Group 4: Others	15 (12.8%)	15 (8.0%)	
Pre-ECMO duration (days) of			
- Hospitalization	10 (8 to 12)	5 (4 to 7)	0.023
- Mechanical ventilation	5 (3 to 7)	2 (2 to 3)	0.013
**Baseline cardiorespiratory parameters**			
Minute ventilation (L/minute)	11.2 (10.6 to 12.0)	10.4 (10.0 to 10.9)	0.038
**Baseline laboratory parameters**			
Lactate (mmol/L)	38 (30 to 47)	23 (20 to 27)	0.002
Lactate dehydrogenase (U/L)	510 (446 to 608)	452 (408 to 499)	0.076
Bilirubin (mg/dL)	1.2 (0.9 to 1.6)	0.9 (0.8 to 1.1)	0.060
ASAT (U/L)	122 (96 to 159)	78 (65 to 96)	0.005
Hemoglobin (g/dL)	9.8 (9.4 to 10.3)	10.9 (10.5 to 11.3)	<0.001
D-dimer (mg/L)	11 (9 to 14)	8 (7 to 10)	0.022
**Procedural characteristics of ECMO treatment**			
Transport-ECMO (no/yes)	34 (29.1%)	99 (52.9%)	<0.001
Red cell transfusions (about 320 mL) per patient	8 (6 to 9)	3 (3 to 4)	<0.001
Plasma transfusions (about 250 mL) per patient	3 (2 to 5)	0 (0 to 1)	0.001
Platelet transfusions (about 270 mL) per patient	1 (0 to 1)	0 (0 to 0)	<0.001
**Laboratory and cardiorespiratory status after one day on ECMO**			
Minute ventilation (L/minute)	5 (5 to 6)	4 (4 to 5)	0.011
Tidal volume (mL)	305 (278 to 332)	278 (262 to 294)	0.097
Norepinephrine infusion (μg/minute/kg)	0.18 (0.13 to 0.25)	0.12 (0.09 to 0.14)	0.041
Blood gas analysis			
- FiO_2_ (%)	65 (60 to 68)	55 (52 to 58)	<0.001
- PaO_2_/FiO_2_ (mmHg)	122 (112 to 133)	146 (137 to 155)	<0.001
Laboratory parameters			
- Lactate (mmol/L)	36 (27 to 47)	24 (21 to 28)	0.002
- C-reactive protein (mg/L)	162 (143 to 180)	214 (196 to 231)	<0.001
- Bilirubin (mg/dL)	1.9 (1.5 to 2.3)	1.1 (1.0 to 1.3)	0.015
- Lactate dehydrogenase (U/L)	584 (502 to 717)	511 (462 to 573)	0.054
- International normalized ratio	1.38 (1.32 to 1.48)	1.27 (1.24 to 1.31)	0.002

Continuous variables are shown as median (95% confidence interval), categorical variables as *n* (%). Differences for survivors and non-survivors are shown for *P* <0.1. A wider comparison can be seen in Additional file 4. ^a^Immunocompromised state included hematological malignancies, solid tumors, solid organ transplantation, high-dose or long-term corticosteroid or other immunosuppressive therapy, or human immunodeficiency virus infection. ^b^Group 1: primary lung failure, including bacterial, viral, fungal or aspiration pneumonia; Group 2: extra-pulmonary sepsis with secondary lung injury; Group 3: multiple trauma with ARDS; Group 4: other pathologies, including near drowning, chronic lung diseases, such as lung fibrosis and lung transplantation. ASAT, aspartate aminotransferase; ECMO, extracorporeal membrane oxygenation; FiO_2_,fraction of inspired oxygen; PaO_2_/FiO_2_, ratio of partial arterial oxygen pressure/fraction of inspired oxygen; SOFA, Sequential Organ Failure Assessment.

### Performance of external risk prediction models in our population

**Figure 1:**
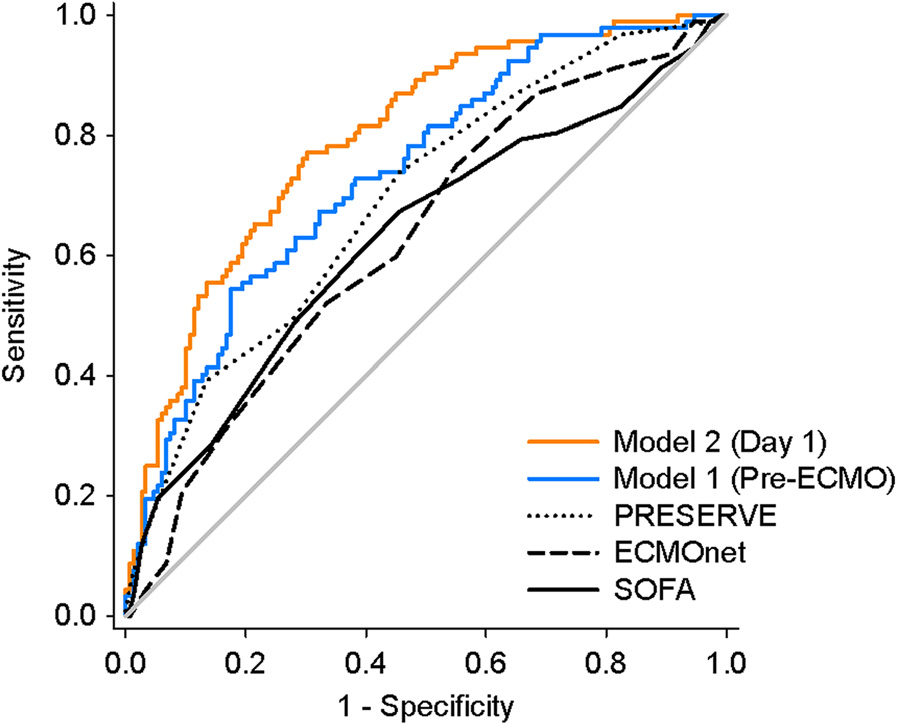
**Comparison of the receiver-operating curves for all risk prediction tools (n = 241).** Neither the ECMOnet nor the PRESERVE score had significantly better discrimination compared to the SOFA score (*P* = 0.67 and 0.25, respectively). Model 1 improved discrimination compared to the SOFA and the ECMOnet score (*P* = 0.03 and 0.009, respectively). Addition of parameters available one day after ECMO initiation further enhanced discrimination compared to both Model 1 and the PRESERVE score (*P* = 0.03 and *P* = 0.003, respectively). Further statistical comparison is given in Table 2. ECMO, extracorporeal membrane oxygenation; SOFA, Sequential Organ Failure Assessment.

Both the ECMOnet and PRESERVE scores showed limited discrimination (AUC <0.70) and poor goodness-of-fit in our population (Table 2). Recalibration of the predictor variables of the ECMOnet score revealed substantial differences in the coefficients of the validation and original study cohorts (data not shown). Discrimination was not improved by recalibration. The proposed cut-off score for mortality >4.5 yielded a sensitivity and specificity of 58% and 53%, respectively. The optimal cut-off for mortality in our population was >5.0, giving a sensitivity and specificity of 52% and 65%, respectively. For the PRESERVE score, the observed mortality rates for score classes 0 to 2, 3 to 4, 5 to 6 and ≥7 showed an increasing trend as in the original study; however, the rates were higher for the lower-risk groups and lower for the higher-risk groups in the present study population (Table 3). PSEP was reduced from 0.81 in the original analysis to 0.53, although the overall mortality rates were similar. Recalibration gave minimal differences in the coefficients of the validation and original study cohort (data not shown). Neither the ECMOnet nor the PRESERVE score showed enhanced discrimination compared to the SOFA score (*P* = 0.67 and *P* = 0.25, respectively, Figure 1).

**Table 2:** Comparison of mortality prediction models in the study population (number = 304)

**Model**	**Valid n**	**AUC**	***P-*** **value**^**a**^	**Hosmer-Lemeshow test *P-*** **value**
**Pre-ECMO prediction**				
SOFA score	303	0.611 (0.544-0.678)	0.027	<0.0001^b^
ECMOnet score	280	0.604 (0.537-0.671)	0.009	0.0002^b^
PRESERVE	289	0.685 (0.623-0.748)	0.12	0.004^b^
Model 1	284	0.746 (0.689-0.804)	Reference	0.50
**Day 1 prediction**				
Model 2	263	0.786 (0.730-0.843)	0.026	0.73

^a^Pairwise comparisons of AUCs with Model 1 using DeLongs method (n = 241) [12]. ^b^Modified Hosmer-Lemeshow test comparing grouped mortality risk against the deciles of Model 1 predictions. AUC, area under receiver operating characteristics curve; ECMO, extracorporeal membrane oxygenation; SOFA, Sequential Organ Failure Assessment.

**Table 3:** Performance of the PRESERVE score in the original and validation cohorts

**PRESERVE score class**	**Original study population**		**UKR ECMO registry**	
	**Number (%)**	**Mortality rate up to six months post-ICU**	**Number (%)**	**In-hospital mortality rate**
0 to 2	34 (25.0%)	3%	35 (12.1%)	11%
3 to 4	38 (27.9%)	21%	90 (31.1%)	28%
5 to 6	26 (19.1%)	46%	97 (33.6%)	40%
≥7	38 (27.9%)	84%	67 (23.2%)	64%
Total	136 (100.0%)	40%	289 (100.0%)	38%
PSEP		81%^a^		53% (95% CI 37–69)

^a^The 95% CI could not be calculated. CI, confidence interval; ECMO, extracorporeal membrane oxygenation; PSEP, prognostic separation index; UKR, University Medical Center Regensburg.

### Novel risk prediction models

#### ***Model 1***

The final pre-ECMO mortality prediction model contained five variables (Table 4). A plot of observed mortality against increasing deciles of predicted mortality risk showed good predictive ability, although the model tended to overestimate mortality in patients with predicted risk between 18% and 23% (third decile group, Additional file 5). The prediction model showed acceptable discrimination (AUC >0.70 but <0.80), a good goodness-of-fit (HL test, *P* = 0.50) and had a shrinkage factor of 0.83.

**Table 4:** Novel mortality prediction models for ALF-patients receiving ECMO support

	**Coefficient**	**OR**	**95% CI**
**Model 1 (pre-ECMO)**			
Age (per five years)	0.176	1.193	(1.148 to 1.239)
Immunocompromised state	0.958	2.605	(1.316 to 5.158)
Minute ventilation (L/minute)	0.098	1.103	(1.014 to 1.199)
Pre-ECMO hemoglobin (g/dL)	-0.182	0.834	(0.728 to 0.954)
Pre-ECMO lactate (mmol/L)	0.013	1.013	(1.004 to 1.023)
Intercept	-2.083		
**Model 2 (Day 1)**			
Age (per five years)	0.184	1.202	(1.148 to 1.258)
Immunocompromised state	1.093	2.984	(1.394 to 6.391)
Minute ventilation (L/minute)	0.137	1.147	(1.030 to 1.276)
Pre-ECMO hemoglobin (g/dL)	-0.208	0.812	(0.696 to 0.947)
Day 1 FiO_2_ (per 10%)	0.264	1.302	(1.232 to 1.376)
Day 1 fibrinogen (mg/dL)	-0.002	0.998	(0.996 to 0.999)
Day 1 norepinephrine (μg/minute/10 kg)	0.159	1.172	(0.980 to 1.401)
Day 1 C-reactive protein (mg/L)	-0.004	0.996	(0.992 to 0.999)
Intercept	-1.893		

ALF, acute lung failure; CI, confidence interval; ECMO, extracorporeal membrane oxygenation; FiO_2_, fraction of inspired oxygen; OR, odds ratio.

##### 

**Comparison of pre-ECMO prediction models** The mortality risk predicted by the external scores did not adequately fit with the observed mortality (modified HL test, *P* <0.01 for all scores, Table 2). By analysis of AUC, Model 1 showed enhanced discrimination compared to the SOFA and ECMOnet score (*P* = 0.03 and *P* = 0.01, respectively, Table 2, Figure 1). However, it did not improve discrimination compared to the PRESERVE score (*P* = 0.12).

#### ***Model 2***

Addition of day-1 parameters resulted in a predictive model based on eight variables (Table 4). Model 2 showed good predictive ability, acceptable discrimination and adequate goodness-of-fit (Table 2). The calibration plot showed good agreement between predicted and observed probability (Additional file 5). The shrinkage factor was 0.87. Model 2 had significantly better predictive ability than Model 1 (LR-test: *P* <0.001) and showed enhanced discrimination (Figure 1) compared both to Model 1 and the PRESERVE score (*P* = 0.03 and *P* = 0.003, respectively). In a separate nested analysis (n = 236), Model 2 (AUC: 0.794 (0.736 to 0.853)) also showed a better discriminative ability compared to the SOFA (AUC: 0.588 (0.512 to 0.665)), ECMOnet (AUC: 0.609 (0.536 to 0.683)) and PRESERVE (AUC: 0.699 (0.629 to 0.768)) scores calculated using day 1 data (*P* <0.001, *P* <0.001 and 0.01, respectively).

Classification of patients into three respective risk groups revealed good agreement between mean predicted and observed group mortality risks for both Models 1 and 2 (Table 5).

**Table 5:** Clinical validation of novel mortality scores

**Predicted risk**	**N**	**Mean predicted risk**	**Observed mortality risk**	**Clinical validation**	**PSEP (95% CI)**
**Model 1**					57 (31 to 84)
<40%	159	22.7%	24.5%	75.5%^a^	
40% to 80%	114	56.2%	54.5%		
>80%	11	86.6%	81.8%	81.8%^b^	
Total population			38.7%		
**Model 2**					61 (43 to 80)
<40%	148	15.7%	19.6%	80.4%^a^	
40% to 80%	94	56.9%	57.4%		
>80%	21	87.1%	81.0%	81.0%^b^	
Total population			38.0%		

^a^Negative predictive value for the low-risk group (<40%). ^b^Positive predictive value for the high-risk group (>80%). CI, confidence interval; n, number of patients in risk group; PSEP, prognostic separation index.

## Discussion

In this large study regarding mortality prediction in ALF patients with vvECMO support, we found that both the PRESERVE and ECMOnet score were suboptimal to predict mortality accurately in our population. Two novel risk models were developed showing improved predictive ability. The addition of parameters available the first day after ECMO initiation enhanced mortality prediction. The combination of predictors found to be associated with the most optimal risk prediction in this study add evidence to the hypothesis that high comorbidity and unresponsive respiratory failure are important determinants of mortality following vvECMO support.

### Pre-existing tools for predicting mortality

The SOFA score was designed for and has become well-integrated in clinical practice as an easily available bedside tool to evaluate organ failure/dysfunction over time in ICU patients. As recently described, a high pre-ECMO SOFA score has been associated with a higher mortality risk [13].

The ECMOnet score is an additive score (range 0 to 10) based on baseline characteristics of 60 patients with severe ARDS due to suspected or confirmed H1N1-influenza virus infection [6]. In our study population, the ECMOnet score did not show better discrimination than the SOFA score. Comparison of original and recalibrated estimates revealed considerable differences between the study populations, but continued poor discrimination after recalibration indicated that the combination of predictors was not useful in our study population. The ECMOnet score has been externally validated twice in patient populations with a similar profile as the original study [6,14]. Recent results indicate that patients receiving ECMO due to influenza-A virus infection have a lower mortality than patients presenting with other causes of ALF [13]. In addition, the ECMOnet study excluded patients with pre-ECMO mechanical ventilation >7 days, leaving a patient population that may have a more favorable risk profile [4]. Although a more homogenous study population improves performance and stability of a risk prediction model, this may also limit its general applicability.

The PRESERVE score was developed in a multicenter study comprising 140 patients with severe ARDS. The additive score (range 0 to 12) consists of eight predictors. To our knowledge, this study is the first to externally validate the score. Minimal differences in predictor distributions as well as similar overall mortality rates support the comparability of the original and validation study populations. There was a clear linear trend in the risk of mortality across increasing subgroups of PRESERVE score. However, the observed differences in the subgroup mortality rates and the reduction in PSEP may be related to overfitting from the original analysis [11]. Again, in our population the PRESERVE score did not show better discrimination than the SOFA score.

### Novel risk models

Based on the confirmed usefulness of the established scores, we identified three strengths in our study which justify our attempt to develop a new risk model: First, the present study (n = 304) is the largest study investigating mortality prediction in ALF patients receiving vvECMO support. Second, the availability of a broad range of variables potentially related to ECMO outcome increases the chances of finding an improved combination of predictors for mortality following vvECMO. Third, the rigor of variable selection and the statistical methods applied have been designed to avoid overfitting the models to the study population [15-17].

The final models described in the present study represent combinations of variables that contain the most useful information to predict ECMO mortality. The models are not designed to reflect causal relationships between single predictors and outcome, which would only be possible if all factors that influence mortality were known. The included predictors may not play causal roles themselves, but carry information from one or several other predictors and thus represent markers for other causal relationships.

The composition of predictors in Models 1 and 2 underline the importance of the patient’s underlying health condition and regenerative capacity for prediction of ECMO outcome. Advanced age and chronic immunosuppression, both associated with reduced functional reserves, high comorbidity and reduced ability to recover, have been consistently associated with increased mortality [7,9,18]. In accordance with previous findings, we found the necessity of high minute ventilation before ECMO to be an important predictor of mortality [9]. A high pre-ECMO serum lactate concentration could indicate tissue hypoxia with subsequent metabolic acidosis and severe organ injury. Parallel to reduced hematocrit described in the ECMOnet study, low pre-ECMO hemoglobin concentrations were associated with an increased mortality risk. Possible causes of pre-ECMO anemia include hospital-acquired anemia, iron deficiency anemia or chronic disease. Preoperative anemia has repeatedly been described as an independent risk factor for increased mortality in cardiac as well as noncardiac surgery [19,20]. The harmful effect of anemia is greater than the increased risk explained by the need for transfusion [20]. Thus, associated comorbid conditions may confound the role of anemia as a risk factor.

The improved performance with Model 2 indicates that ECMO relieves patients from acute respiratory and hemodynamic stress and provides time to recover already within the first 24 hours. In patients with severe hypoxemia or high cardiac output, where ECMO may not improve gas exchange sufficiently to decrease aggressiveness of mechanical ventilation, continued invasive ventilation with high volume and FiO_2_ may bring about a vicious cycle with ventilator-induced lung injury [4]. Thus, sparse reduction or continued need for a high FiO_2_ and norepinephrine on day 1 may indicate patients with unresponsive respiratory failure despite ECMO.

Furthermore, day 1 plasma concentrations of fibrinogen and C-reactive protein (CRP) were identified as important predictors of ECMO outcome. Surprisingly, low CRP was associated with increased risk of mortality. This might be related to the ability to activate the immune system in order to defend against on-going infection and trigger recovery mechanisms. Whereas survivors generally had higher CRP before and one day after ECMO implantation, they had lower CRP concentrations post-ECMO, leading to the hypothesis that survivors were able to quickly activate an effective immune response. The lower CRP in non-survivors, on the other hand, might indicate immune exhaustion or liver failure, with a failing attempt to bring about an effective response in order to establish control of their acute illness.

The protective effect seen with higher fibrinogen concentrations may have several explanations. First, it may be another sign of an effective immune response, supported by the role of fibrinogen as an acute phase reactant. Given that both fibrinogen and CRP remained significant, they both provide information in addition to that from the other factor, even if the two may be somewhat correlated. Second, low fibrinogen levels are also associated with bleeding disorders, large-volume blood transfusions and disseminated intravascular coagulation, which are all associated with higher mortality. Fibrinogen may thus be an informative marker replacing the need for many individual markers and may contain information from several functions that affect outcome following ECMO support.

All together, the parameters from Model 2 enhanced discrimination between patients with high regenerative capacity and reversible disease from those with poor health condition, reduced functional reserves and intractable illness.

### Challenges related to mortality prediction in vvECMO patients

This study illustrates the difficulties in creating a robust model for predicting mortality following vvECMO. Model 1 showed improved goodness-of-fit and somewhat improved discrimination; however, the discriminative ability was not significantly enhanced from the PRESERVE score. ECMO patients have a large heterogeneity in disease and health conditions, and behind ‘mortality of all causes’ there is a large diversity in underlying causality. Patients have different diagnoses, time courses, ages, comorbidities, different sites and types of infection, and different degrees of physiological dysfunction [21]. Therefore, inclusion of day1 parameters seemed a promising approach to better prediction. The external scores calculated on day 1 data did not provide adequate risk prediction, supporting the usefulness of our new model.

It would be of interest to develop a prediction model for use later during the course of ECMO, for example on day 7. Such a model might aid in the decision to wean the patient from ECMO. However, due to smaller patient numbers caused by earlier weaning or death, a preliminary day 7 model for the remaining 193 patients (126 survivors and 67 non-survivors) showed wide CIs for the odds ratios and was not significantly better at predicting mortality than the day 1 model (*P* = 0.25, data not shown). Thus, it seems that a substantially larger study would be needed. Furthermore, any model aimed at predicting withdrawal of futile therapy has to be extremely accurate in order not to lead to erroneous decisions. From our present experience with 350 patients, we have observed previously unthought-of capacities of injured lungs to recover.

In this study, different statistical approaches were explored and compared in order to derive the best and most robust prediction model. While previous models have based their variable selection on univariate analyses, we employed a method combining clinical experience and judgment with computer-based statistics. Nevertheless, the inhomogeneous study population makes overfitting a persisting challenge and the ability of a predictive model to estimate the prognosis of individual patients with high accuracy remains limited. The usefulness of a prediction model rather lies in the assistance it may provide for the discrimination between higher- and lower-risk patients in order to help identify potential candidates for ECMO support and give guidance for rational and ethical resource utilization [6,11]. We, therefore, categorized patients into three groups with significantly different probabilities of survival. It may be argued that our defined high-risk group was very small, but we kept this cut-off because of its potential clinical usefulness. Categorization of patients may also be helpful for future external and internal validations in order to compare institutional variations in indications and practice of ECMO. However, although the risk score may function as a useful supplementary tool for clinicians, thorough clinical evaluation on a case-to-case basis still remains the cornerstone of ECMO handling.

### Study limitations

Conventional rescue therapies, such as prone positioning and use of neuromuscular blockade, were not registered in all patients in the UKR database. A part of our study population dates back to 2008 when the advantages of prone positioning were not universally accepted and neuromuscular blockade was only rarely used in Germany. Hence, the quality of conventional treatment which may be associated with mortality irrespective of the other factors included in the model could not be evaluated in this study. In the calculation of the PRESERVE scores, all patients were assigned to ‘no prone positioning pre-ECMO.’ However, this may contribute to overestimating mortality risk with PRESERVE in our patient population. Furthermore, the PRESERVE score used mortality six months post-ICU discharge as the end-point, while the present study only followed patients up to hospital discharge. Mid- and long-term outcomes are important in ARDS patients receiving vvECMO support. Unfortunately, in the past, long-term outcomes of our patients and health-related quality of life were not reevaluated, as many patients were retrieved from distant hospitals after our team implanted ECMO as a rescue procedure to allow transport to our center. We plan to carry out a follow-up study to evaluate the validity of the presented models in the prediction of mid- and long-term mortality.

Missing observations due to incomplete documentation for some laboratory and respiratory parameters reduced the effective sample size in the statistical analyses. Bias due to cohort selection or missing data in the presented analyses seems unlikely since the overall mortality rate was constant in all subsamples with complete information for the different parts of the study. Since validation through bootstrap methods is preferred over data splitting methods [17], we used the whole patient cohort for model development. However, external validation is a crucial step before these models can be applied in clinical practice.

## Conclusions

Pre-existing tools for predicting outcome following vvECMO in patients with ALF have been of limited usefulness. The present study provides two novel risk models designed to help assist in defining treatment decision and further handling of ECMO. Markers of associated comorbidity, a poor pre-ECMO health condition and unresponsive respiratory failure were consistently associated with increased mortality. Mortality prediction improved the day following ECMO initiation, when ECMO has reduced acute stress and provided time to rest. Nevertheless, due to high variability among patients, predictive models can only act as supplementary tools and do not replace the role of clinical evaluation for indication and continuation of ECMO.

## Key messages

• Established risk assessment tools for adult patients with severe acute lung failure on vvECMO are of limited usefulness.

• Large patient heterogeneity makes generalized pre-ECMO mortality prediction a persisting challenge, despite use of robust statistical methods.

• Addition of variables measured one day after ECMO initiation improves mortality prediction.

• Risk prediction tools function as a useful supplement to guide clinical decision-making, although clinical evaluation remains the cornerstone of ECMO practice.

## Endnote

^a^Available at: http://www.r-project.org. Accessed June 15, 2013.

## Abbreviations

ALF: acute lung failure; ARDS: acute respiratory distress syndrome; AUC: area under receiver-operating characteristics curve; CI: confidence interval; CRP: C-reactive protein; FiO2: fraction of inspired oxygen; HL test: Hosmer-Lemeshow test; ICU: intensive care unit; LR test: likelihood ratio test; PaO2/FiO2: ratio of partial arterial pressure of oxygen/fraction of inspired oxygen; PRESERVE: PRedicting dEath for SEvere ARDS on VV-ECMO; PSEP: prognostic separation index; SOFA: Sequential Organ Failure Assessment; UKR: University Medical Center Regensburg; vvECMO: veno-venous extracorporeal membrane oxygenation.

## Competing interests

CS has been a consultant for Maquet Cardiopulmonary Care Germany. AP and TM received travel support from Maquet Cardiopulmonary Care Germany. TB is member of the advisory board of Novalung, Heilbronn Germany and he received honoraria. None of the other authors have a financial relationship with a commercial entity that has an interest in the subject of the presented manuscript or other conflicts of interest to disclose.

## Authors’ contributions

TBE made substantial contributions to the conception and design of the study, data analysis, statistical analyses and interpretation of data, and wrote and revised the manuscript. VV made substantial contributions to the conception and design of the study, statistical analyses and interpretation of data, and has been involved in drafting the manuscript and revising it critically for important intellectual content. AP and TM made substantial contributions to the conception and design of this study, acquisition and interpretation of data, and have been involved in drafting the manuscript and revising it critically for important intellectual content. AW made substantial contributions to the conception and design of the study, interpretation of data and has been involved in revising the manuscript critically for important intellectual content. ML, MF, CS and TB have made substantial contributions to the acquisition of data, interpretation of data and have been involved in revising the manuscript critically for important intellectual content. All authors read and approved the final manuscript.

## Supplementary Material

Additional file 1 Detailed description of data collection and statistical methods. Click here for file

Additional file 2 **Data on external scores.** Information about the calculation of the SOFA, ECMOnet and PRESERVE scores validated in the study. Click here for file

Additional file 3 **Variable selection and modelling.** An overview showing the three-step variable selection procedure used in order to derive two parsimonious models designed for predicting mortality in patients with severe lung failure before and one day after ECMO implementation, respectively. Click here for file

Additional file 4 **Comparison of survivors and non-survivors.** A detailed comparison between survivors and non-survivors with regard to baseline clinical and proce characteristics, as well as cardiorespiratory and laboratory parameters measured pre-ECMO and regularly throughout ECMO support. Click here for file

Additional file 5 **Observed versus predicted probabilities estimated by Model 1 and Model 2.** A figure comparing the relationship between observed and predicted mortalities as well as model calibration for Models 1 and 2. Click here for file
