# Supplementary material for: Prediction of mortality in adult patients with severe acute lung failure receiving veno-venous extracorporeal membrane oxygenation: a prospective observational study
Source: Crit Care. 2014 Apr 9;18(2):R67. doi: 10.1186/cc13824 (PMC4057201; doi:10.1186/cc13824)
Supplement: Additional file 1 — Detailed description of data collection and statistical methods. [file cc13824-S1.pdf]

ADDITIONAL FILE 1: Detailed description of data collection and statistical methods

Data collection

The UKR database comprises a wide range of pre-, intra- and post-ECMO characteristics that have been collected as a standard routine by the ECMO-specialized team. Pre-ECMO characteristics included among others age, gender, body mass index (BMI), primary diagnosis and relevant comorbidity. Blood gas analyses, ventilator and ECMO settings, laboratory parameters, hemodynamic parameters and vasopressor therapy use were registered regularly before, during and after ECMO implantation until the second day after weaning from ECMO. ICU events, including patient-related complications as well as technical problems were documented continuously. Duration of ECMO and mechanical ventilation support, ICU and hospital lengths-of-stay were registered.

Model 1: Pre-ECMO mortality prediction

Variable selection for prediction of in-hospital death followed a 3-step-procedure (see Additional file 2): A liberal pre-selection of potential predictors was based on literature review, clinical experience and hypotheses of potential influences on ECMO outcome. From this selection, irrelevant variables were eliminated using random forest analysis, i.e. using bootstrapping (n=2000) and classification of variables into best explanatory sets to reduce the effect of random variability in the original dataset [1]. The remaining predictors were further investigated with multivariate logistic regression analysis. The models were tested for predefined interactions, linearity in the logit for continuous variables and overly-influential observations. A parsimonious model was obtained using limited backwards stepdown, where variables were retained according to Akaike's Information Criterion. Subsequent model calibration and validation was performed as described in the original article.

Comparison of pre-ECMO prediction models

For comparison of goodness-of-fit across the different risk scores, the additive scores were rescaled to a value between 0 and 1 by division with their respective maximum score. A modified Hosmer-Lemeshow test was then used to evaluate the goodness-of-fit of the external risk scores across the risk groups defined by Model 1. The test assesses the agreement of the observed mortality rate to the expected mortality rate in subgroups of the model population. If the p-value of the test >0.05, there is no significant difference between observed and expected mortality rates, and the model is said to be well-calibrated. The discriminative abilities of the different models were compared pairwise applying Delong's method for non-parametric correlated AUCs [2].

Evaluating the clinical value of a ECMO prediction model

In addition to statistically assessing the performance of the model, the clinical usefulness of the prediction models was evaluated by classification of patients into groups with different prognoses: A lower-risk group (predicted probability <40%), an intermediate (40-80%) and a higher-risk group (>80%). These groups were defined by clinical discretion, as recommended by Altman and Royston [3]. The size of prognostic information was quantified by predicted separation (PSEP) methods; assessing the spread of probabilities in the lower- and higher risk group: The index was calculated as the difference between the predicted probability of dying for a patient in the group with the worst and best diagnosis, respectively. PSEP calculations are also useful for future external validation, as a

reduction in separation in future study cohorts may indicate over-optimism in the predictive ability of the model [3].

Since it is conceivable that a prediction model based on a heterogeneous study population cannot yield perfect fit, we further calculated negative and positive predictive values for the lowest and highest risk groups, respectively:

Negative predictive value: Number of observed survivors/Number of predicted survivors

Positive predictive value: Number of observed in-hospital deaths/Number of predicted in-hospital deaths

References

1. Strobl C, Malley J, Tutz G: **An introduction to recursive partitioning: rationale, application, and characteristics of classification and regression trees, bagging, and random forests**. *Psychological methods* 2009, **14**(4):323-348.
2. DeLong ER, DeLong DM, Clarke-Pearson DL: **Comparing the areas under two or more correlated receiver operating characteristic curves: a nonparametric approach**. *Biometrics* 1988, **44**(3):837-845.
3. Altman DG, Royston P: **What do we mean by validating a prognostic model?** *Stat Med* 2000, **19**(4):453-473.
