# Supplementary material for: Prediction of mortality in adult patients with severe acute lung failure receiving veno-venous extracorporeal membrane oxygenation: a prospective observational study
Source: Crit Care. 2014 Apr 9;18(2):R67. doi: 10.1186/cc13824 (PMC4057201; doi:10.1186/cc13824)
Supplement: Additional file 2 — Data on external scores. Information about the calculation of the SOFA, ECMOnet and PRESERVE scores validated in the study. [file cc13824-S2.pdf]

**ADDITIONAL FILE 2: Data on external scores**

Calculation of the SOFA score

Table 1: The SOFA score

| SOFA score | 0 | 1 | 2 | 3 | 4 |
|---|---|---|---|---|---|
| **Respiration**<br>$PaO_2/FIO_2$ (mm Hg) | >400 | <400 | <300 | <200[a] | <100[a] |
| **Coagulation**<br>Platelets $x10^3/mm^3$ | >150 | <150 | <100 | <50 | <20 |
| **Liver**<br>Bilirubin (mg/dL) | <1.2 | 1.2-1.9 | 2.0-5.9 | 6.-11.9 | >12.0 |
| **Cardiovascular**[b]<br>Hypotension | No hypotension | MAP <70 | Dopamine ≤5 or dobutamine (any dose) | Dopamine >5 or norepinephrine /epinephrine ≤0.1 | Dopamine >15 or norepinephrine/ epinephrine >0.1 |
| **CNS**[c]<br>Glasgow Coma Score | 15 | 13-14 | 10-12 | 6-9 | <6 |
| **Renal**<br>Creatinine (mg/dL) or urine output (mL/day) | <1.2 | 1.2-1.9 | 2.0-3.4 | 3.5-4.9 or <500 | >5.0 or <200 |

*MAP* mean arterial pressure, *CNS* central nervous system.
[a]with respiratory support
[b]vasoactive mediations administered for at least 1 hr (doses are given in ug/kg/min)
[c]as we do not know the "real" Glasgow Coma Scale in intubated patients, the Glasgow Coma Scale was set to 15 (=SOFA score 0) for all patients.

*Reprinted with kind permission from Springer Science+Business Media: Intensive care medicine, The SOFA (Sepsis-related Organ Failure Assessment) score to describe organ dysfunction/failure. On behalf of the Working Group on Sepsis-Related Problems of the European Society of Intensive Care Medicine, Vol. 22, 1996, pp. 707-710, Vincent JL, Moreno R, Takala J, Willatts S, De Mendonca A, Bruining H, Reinhart CK, Suter PM, Thijs LG, Table 3.*

Calculation of the ECMOnet score

In accordance with the original manuscript, the ECMOnet score for each individual was calculated with the following formula where $ps_i$ is the partial score assigned to each of the 5 parameters as given in Table 2:

$$ECMOnet\ score = \left(\sum_{i=1}^{5} ps_i\right) - 1$$

Table 2: The ECMOnet score

| **PARAMETER** | **PARTIAL SCORE ($ps_i$)** |
|---|---|
| *1) PreECMO hospital length of stay, days* | |
| ≤ 3 | 0.5 |
| 4 - 7 | 1 |
| 8 - 11 | 1.5 |
| > 11 | 2 |
| *2) Bilirubin, mg/dl* | |
| ≤ 0.15 | 0 |
| 0.16 - 0.65 | 0.5 |
| 0.66 - 1.15 | 1 |
| 1.16 - 1.65 | 1.5 |
| 1.66 - 2.15 | 2 |
| > 2.15 | 2.5 |
| *3) Creatinine, mg/dl* | |
| ≤ 0.5 | 0 |
| 0.51 - 0.80 | 0.5 |
| 0.81 - 1.10 | 1 |
| 1.11 - 1.40 | 1.5 |
| 1.41 - 1.70 | 2 |
| 1.71 - 2.00 | 2.5 |
| 2.01 - 2.30 | 3 |
| > 2.30 | 3.5 |
| *4) Hematocrit, %* | |
| > 40 | 0.5 |
| 36 - 40 | 1 |
| 31 - 35 | 1.5 |
| ≤ 30 | 2.0 |
| *5) Mean arterial pressure, mmHg* | |
| > 90 | 0 |
| 61 - 90 | 0.5 |
| ≤ 60 | 1 |

*ECMO* extracorporeal membrane oxygenation

*Reprinted with kind permission from Springer Science+Business Media: Intensive care medicine, Predicting mortality risk in patients undergoing venovenous ECMO for ARDS due to influenza A (H1N1) pneumonia: the ECMOnet score, Vol. 39, 2013, pp. 275-281, Pappalardo F, Pieri M, Greco T, Patroniti N, Pesenti A, Arcadipane A, Ranieri VM, Gattinoni L, Landoni G, Holzgraefe B, Beutel G, Zangrillo A, Italian ECMOnet, Table 2.*

<u>Calculation of the PRESERVE score</u>

Table 3: The PRESERVE score calculated with parameters available at the time of decision to initiate ECMO

| **Parameter** | **Score** |
|---|---|
| Age (years) | |
| <45 | 0 |
| 45–55 | 2 |
| >55 | 3 |
| Body mass index >30 | −2 |
| Immunocompromised | 2 |
| SOFA >12[a] | 1 |
| MV >6 days | 1 |
| No prone positioning before ECMO | 1 |
| PEEP < 10 cm $H_2O$ | 2 |
| Plateau pressure >30 cm $H_2O$ | 2 |
| Total score[c] | 0–12 |

*ECMO* extracorporeal membrane oxygenation, *ICU* intensive care unit, *MV* mechanical ventilation, *PEEP* positive end-expiratory pressure, *PRESERVE* PRedicting dEath for SEvere ARDS on VV-ECMO, *SAPS II* simplified acute physiology score, *SOFA* sepsis-related organ failure assessment

[a]Immunocompromised status included hematological malignancies, solid tumors, solid organ transplantation, high-dose or long-term corticosteroid and/or immunosuppressant use, or human immunodeficiency virus infection

[b]SOFA score was preferred over SAPS II (excluding the age component) for simpler use of the score at the bedside

[c]Higher score indicates higher probability of death by 6 months post-ICU discharge; PRESERVE scores −1 and −2 converted to 0 for simplification

*Reprinted with kind permission from Springer Science+Business Media: Intensive care medicine, The PRESERVE mortality risk score and analysis of long-term outcomes after extracorporeal membrane oxygenation for severe acute respiratory distress syndrome, Vol. 39, 2013, pp. 1704-1713, Schmidt M, Zogheib E, Roze H, Repesse X, Lebreton G, Luyt CE, Trouillet JL, Brechot N, Nieszkowska A, Dupont H, Ouattara A, Leprince P, Chastre J, Combes A, Table 4.*
