# Supplementary material for: Prediction of mortality in adult patients with severe acute lung failure receiving veno-venous extracorporeal membrane oxygenation: a prospective observational study
Source: Crit Care. 2014 Apr 9;18(2):R67. doi: 10.1186/cc13824 (PMC4057201; doi:10.1186/cc13824)
Supplement: Additional file 3 — Variable selection and modelling. An overview showing the three-step variable selection procedure used in order to derive two parsimonious models designed for predicting mortality in patients with severe lung failure before and one day after ECMO implementation, respectively. [file cc13824-S3.pdf]

**ADDITIONAL FILE 3: Variable selection and modeling.**

An overview showing the 3-step variable selection procedure used in order to derive two parsimonious models designed for predicting mortality in patients with severe lung failure before and one day after ECMO implementation, respectively. The table shows the variables retained at each step.

| Clinical characteristics | Laboratory parameters | | Respiratory parameters/blood gas | |
|---|---|---|---|---|
| Pre-ECMO | Pre-ECMO | Day 1 | Pre-ECMO | Day 1 |
| **Step 1: Liberal variable selection based on clinical evaluation and literature review** | | | | |
| Age<br>Gender<br>BMI<br>Primary diagnosis (group 1-4)[1]<br>Immunocompromised state[2]<br>SOFA score<br>MV pre-ECMO (days)<br>Hospitalization pre-ECMO (days)<br>CVVHF pre-ECMO<br>Kidney dysfunction pre-ECMO[3] | Lactate<br>Haemoglobin<br>Bilirubin<br>ASAT<br>Creatinine<br>CrCl<br>LDH<br>CRP | ASAT<br>Bilirubin<br>Creatinine<br>LDH<br>CRP<br>D-dimer<br>Fibrinogen<br>INR | Norepinephrine dosage<br>Mean arterial pressure<br>Minute ventilation<br>Tidal volume<br>Pmax<br>$PaO_2/FiO_2$<br>Arterial $pCO_2$<br>Arterial pH<br>Oxygen saturation | Pmax<br>Blood flow<br>Tidal volume<br>$FiO_2$<br>$PaO_2/FiO_2$<br>Norepinephrine dosage<br>Venous saturation<br>Arterial pH<br>Arterial $PaCO_2$<br>Arterial base excess<br>Arterial standard bicarbonate |
| **Step 2: Variables from Step 1 reduced to the following by random forest analysis and variable importance ranking** | | | | |
| Age<br>Immunocompromised state<br>SOFA score<br>MV pre-ECMO (days)<br>Hospitalization pre-ECMO (days)<br>CVVHF pre-ECMO | Lactate<br>Haemoglobin<br>ASAT | Bilirubin<br>CRP<br>Fibrinogen<br>INR | Minute ventilation | $FiO_2$<br>Norepinephrine |
| **Step 3: Variables from Step 2 reduced to the following by multivariate logistic regression modeling with limited backwards stepdown** | | | | |
| Age[4,5]<br>Immunocompromised state[4,5] | Lactate[4]<br>Haemoglobin[4,5] | CRP[5]<br>Fibrinogen[5] | Minute ventilation[4,5] | $FiO_2$[5]<br>Norepinephrine dosage[5] |

ASAT: aspartate-aminotransferase; BMI: body mass index; CrCl: estimated creatinine clearance; CRP: C-reactive protein; CVVHF: continuous veno-venous haemofiltration; ECMO: extracorporeal membrane oxygenation; $FiO_2$: fraction of inspired oxygen; INR: international normalized ratio; LDH: lactate dehydrogenase; MV: mechanical ventilation; Pmax: maximum inspiratory pressure on mechanical ventilation; SOFA: sequential organ failure assessment; $PaCO_2$: partial pressure of carbon dioxide; $PaO_2/FiO_2$: ratio of arterial oxygen pressure/fraction of inspired oxygen

[1]Group 1: primary lung failure, including bacterial, viral, fungal or aspiration pneumonia; Group 2: extra-pulmonary sepsis with secondary lung injury; Group 3: multiple trauma with ARDS; Group 4: other pathologies, including near drowning, chronic lung diseases such as lung fibrosis and lung transplantation.

[2]Immunocompromised state included haematological malignancies, solid tumours, solid organ transplantation, high-dose or long-term corticosteroid or other immunosuppressive therapy, or human immunodeficiency virus infection

[3]Kidney dysfunction defined as continuous veno-venous haemofiltration pre-ECMO or creatinine clearance <90mL/min.

[4]Final variables in Model 1 (pre-ECMO).

[5]Final variables in Model 2 (day 1). Model 2 was built as an extension of Model 1. In this model, pre-ECMO lactate concentration was no longer a significant predictor of mortality in the last step.
