# Supplementary material for: Prediction of mortality in adult patients with severe acute lung failure receiving veno-venous extracorporeal membrane oxygenation: a prospective observational study
Source: Crit Care. 2014 Apr 9;18(2):R67. doi: 10.1186/cc13824 (PMC4057201; doi:10.1186/cc13824)
Supplement: Additional file 4 — Comparison of survivors and non-survivors. A detailed comparison between survivors and non-survivors with regard to baseline clinical and proce characteristics, as well as cardiorespiratory and laboratory parameters measured pre-ECMO and regularly throughout ECMO support. [file cc13824-S4.pdf]

ADDITIONAL FILE 4: Comparison of survivors and non-survivors

**Baseline clinical characteristics**	**Non-survivors (n=117)**	**Survival to discharge (n=187)**	***P*-value**
Age (years)	54 (50-57)	46 (43-48)	<0.001
Female gender	36 (30.8%)	64 (34.2%)	0.53
Body mass index (kg/m^2)	28.8 (27.6-30.2)	28.8 (27.8-29.9)	0.94
Immunocompromised state[1]	37 (31.6%)	26 (13.9%)	<0.001
Lung injury score	3.5 (3.4-3.5)	3.5 (3.4-3.5)	0.90
Sequential organ failure assessment score	13 (12-14)	11 (11-12)	0.001
Continuous veno-venous haemofiltration pre-ECMO	45 (38.5%)	36 (19.3%)	<0.001
Classification of acute lunge injury[2]			0.022
– Group 1: Pulmonary	56 (47.9%)	107 (57.2%)	
– Group 2: Extra-pulmonary	36 (30.8%)	36 (19.3%)	
– Group 3: Trauma	10 (8.6%)	29 (15.5%)	
– Group 4: Others	15 (12.8%)	15 (8.0%)	
Norepinephrine infusion (µg/min/kg)	0.37 (0.29-0.46)	0.33 (0.29-0.39)	0.63
Pre-ECMO duration (days)			
– Hospitalization	10 (8-12)	5 (4-7)	0.023
– Mechanical ventilation	5 (3-7)	2 (2-3)	0.013
**Baseline cardiorespiratory parameters**			
Mean arterial pressure (mm Hg)	71 (68-73)	69 (67-71)	0.28
Minute ventilation (L/min)	11.2 (10.6-12.0)	10.4 (10.0-10.9)	0.038
Tidal volume (mL)	479 (452-503)	480 (459-502)	0.93
Static compliance (mL/cm H_2O)[3]	27.1 (24.9-29.6)	27.2 (25.6-28.8)	0.85
Corrected expiratory volume (L/min)[4]	18.1 (16.7-19.5)	17.2 (16.1-18.4)	0.41
Positive end-expiratory pressure (cm H_2O)	17 (16-18)	16 (16-17)	0.40
Mean airway pressure (cm H_2O)	23 (22-24)	23 (22-24)	0.82
Peak inspiratory pressure (cm H_2O)	36 (35-37)	35 (34-36)	0.24
Arterial blood gas analysis			
– FiO_2 (%)	100 (98-100)	100 (100-100)	0.51
– PaO_2/FiO_2 (mm Hg)	67 (62-73)	69 (65-74)	0.55
– SaO_2 (% saturation)	88 (85-90)	89 (87-90)	0.89
– $PaCO_2$ (mm Hg)	65 (61-69)	68 (64-72)	0.30

– pH	7.24 (7.21-7.27)	7.22 (7.22-7.25)	0.37
– Base excess (mmol/L)	-1.3 (-2.7-0.3)	-1.2 (-2.1- -0.1)	0.88
**Baseline laboratory parameters**			
C-reactive protein (mg/L)	150 (127-170)	163 (140-182)	0.36
Lactate (mmol/L)	38 (30-47)	23 (20-27)	0.002
Lactate dehydrogenase (U/L)	510 (446-608)	452 (408-499)	0.076
Creatinine (mg/dL)	1.4 (1.2-1.6)	1.4 (1.2-1.6)	0.96
Creatinine clearance (mL/min)[5]	89.2 (79.6-100.1)	99.8 (89.2-110.6)	0.38
Bilirubin (mg/dL)	1.2 (0.9-1.6)	0.9 (0.8-1.1)	0.060
ASAT (U/L)	122 (96-159)	78 (65-96)	0.005
Fibrinogen (mg/dL)	523 (471-579)	562 (521-606)	0.24
Leukocytes (/µL)	12.8 (11.1-14.7)	14.1 (12.8-15.5)	0.20
Haemoglobin (g/dL)	9.8 (9.4-10.3)	10.9 (10.5-11.3)	<0.001
Platelets (x10^9/L)	171 (147-195)	185 (167-205)	0.23
International normalized ratio	1.30 (1.24-1.38)	1.23 (1.19-1.28)	0.17
Prothrombin time (sec)	50 (46-55)	48 (45-51)	0.21
D-dimer (mg/L)	11 (9-14)	8 (7-10)	0.022
**Procedural characteristics of ECMO treatment**			
Transport-ECMO (no/yes)	34 (29.1%)	99 (52.9%)	<0.001
ECMO duration (days)	11 (9-13)	10 (9-11)	0.60
Red cell transfusions (~320 mL) per patient	8 (6-9)	3 (3-4)	<0.001
Plasma transfusions (~250 mL) per patient	3 (2-5)	0 (0-1)	0.001
Platelet transfusions (~270 mL) per patient	1 (0-1)	0 (0-0)	<0.001
Continuous veno-venous haemofiltration during ECMO	25 (21.4%)	34 (18.2%)	0.49
**Laboratory and cardiorespiratory status the first day on ECMO**			
Venous oxygen saturation (%)	70 (68-72)	70 (69-72)	0.57
Minute ventilation (L/min)	5 (5-6)	4 (4-5)	0.011
Tidal volume (mL)	305 (278-332)	278 (262-294)	0.097
Blood flow on ECMO (L/min)	2.8 (2.7-3.0)	2.8 (2.7-2.9)	0.89
Norepinephrine infusion (µg/min/kg)	0.18 (0.13-0.25)	0.12 (0.09-0.14)	0.041
Blood gas analysis			
– FiO_2 (%)	65 (60-68)	55 (52-58)	<0.001
– PaO_2/FiO_2 (mm Hg)	122 (112-133)	146 (137-155)	<0.001
– Arterial pH	7.43 (7.41-7.45)	7.44 (7.43-7.46)	0.14
– Arterial $PaCO_2$ (mm Hg)	37 (36-39)	38 (37-39)	0.77

Laboratory parameters			
– C-reactive protein (mg/L)	162 (143-180)	214 (196-231)	<0.001
– Lactate (mmol/L)	36 (27-47)	24 (21-28)	0.002
– Lactate dehydrogenase (U/L)	584 (502-717)	511 (462-573)	0.054
– Creatinine (mg/dL)	1.3 (1.0-1.8)	1.1 (1.0-1.2)	0.21
– Bilirubin (mg/dL)	1.9 (1.5-2.3)	1.1 (1.0-1.3)	0.015
– International normalized ratio	1.38 (1.32-1.48)	1.27 (1.24-1.31)	0.002
**Laboratory and cardiorespiratory analysis during ECMO**			
Minimum arterial pH <7.20	23 (19.8%)	6 (3.2%)	<0.001
Maximum arterial pH >7.55	24 (20.5%)	51 (27.3%)	0.18
Minimum lactate (mmol/L)	15 (12-22)	7 (6-7)	<0.001
Maximum lactate (mmol/L)	87 (70-103)	39 (34-46)	<0.001
Minimum $PaCO_2$ (mm Hg)	30 (29-31)	30 (30-31)	0.55
Maximum $PaCO_2$ (mm Hg)	48 (46-50)	51 (50-52)	0.001
Maximum blood flow on ECMO (L/min)	3.1 (3.0-3.2)	3.3 (3.1-3.4)	0.001
Minimum PaO_2/FiO_2 (mm Hg)	76 (71-81)	101 (95-106)	<0.001
C-reactive protein at end of ECMO (mg/dL)	137 (105-173)	88 (76-101)	0.007

Continuous variables are shown as median (95% confidence interval), categorical variables as *n* (%).

ALAT: alanine aminotransferase; ASAT: aspartate aminotransferase; CVVHF: continuous veno-venous hemofiltration; ECMO: extracorporeal membrane oxygenation; FiO_2: fraction of inspired oxygen; LIS: lung injury score; $PaCO_2$: partial arterial pressure of carbon dioxide; PaO_2/FiO_2: ratio of partial arterial oxygen pressure/fraction of inspired oxygen; SOFA: sequential organ failure assessment

[1]Immunocompromised state included haematological malignancies, solid tumours, solid organ transplantation, high-dose or long-term corticosteroid or other immunosuppressive therapy or human immunodeficiency virus infection.
[2]Group 1: Primary lung failure, including bacterial, viral, fungal or aspiration pneumonia; group 2: Extra-pulmonary sepsis with secondary lung injury; group 3: Multiple trauma with ARDS; group 4: Other pathologies, including near drowning, chronic lung diseases such as lung fibrosis and lung transplantation.
[3]Static compliance of the respiratory system was calculated as tidal volume (mL) divided by difference between peak inspiratory pressure (cm H_2O) and positive end-expiratory pressure (cm H_2O).
[4]The corrected expiratory volume per minute was calculated as the measured minute ventilation (L/min) multiplied by the arterial partial pressure of carbon dioxide (mm Hg) divided by 40 mm Hg.
[5]Creatinine clearance was calculated based on formula from Cockroft and Gault [1].

References:

1. Cockcroft DW, Gault MH: **Prediction of creatinine clearance from serum creatinine**. *Nephron* 1976, **16**(1):31-41.
