# Supplementary material for: Prediction of mortality in adult patients with severe acute lung failure receiving veno-venous extracorporeal membrane oxygenation: a prospective observational study
Source: Crit Care. 2014 Apr 9;18(2):R67. doi: 10.1186/cc13824 (PMC4057201; doi:10.1186/cc13824)

ADDITIONAL FILE 5:

Figure 1: Observed versus predicted probabilities estimated by Model 1 (panels A and B) and Model 2 (panels C and D).

Panels A and C: Calibration plots for Model 1 and 2 respectively. Apparent prediction (small-dotted line) is compared to perfect prediction (spaced line). The bias-corrected line shows prediction after bootstrap (continuous line) and the prediction after correcting with the estimated shrinkage factor (0.83 and 0.87 for Model 1 and Model 2, respectively, black dots).
Panels B and D: Observed probabilities against decile groups of predicted risk with Model 1 and 2, respectively.

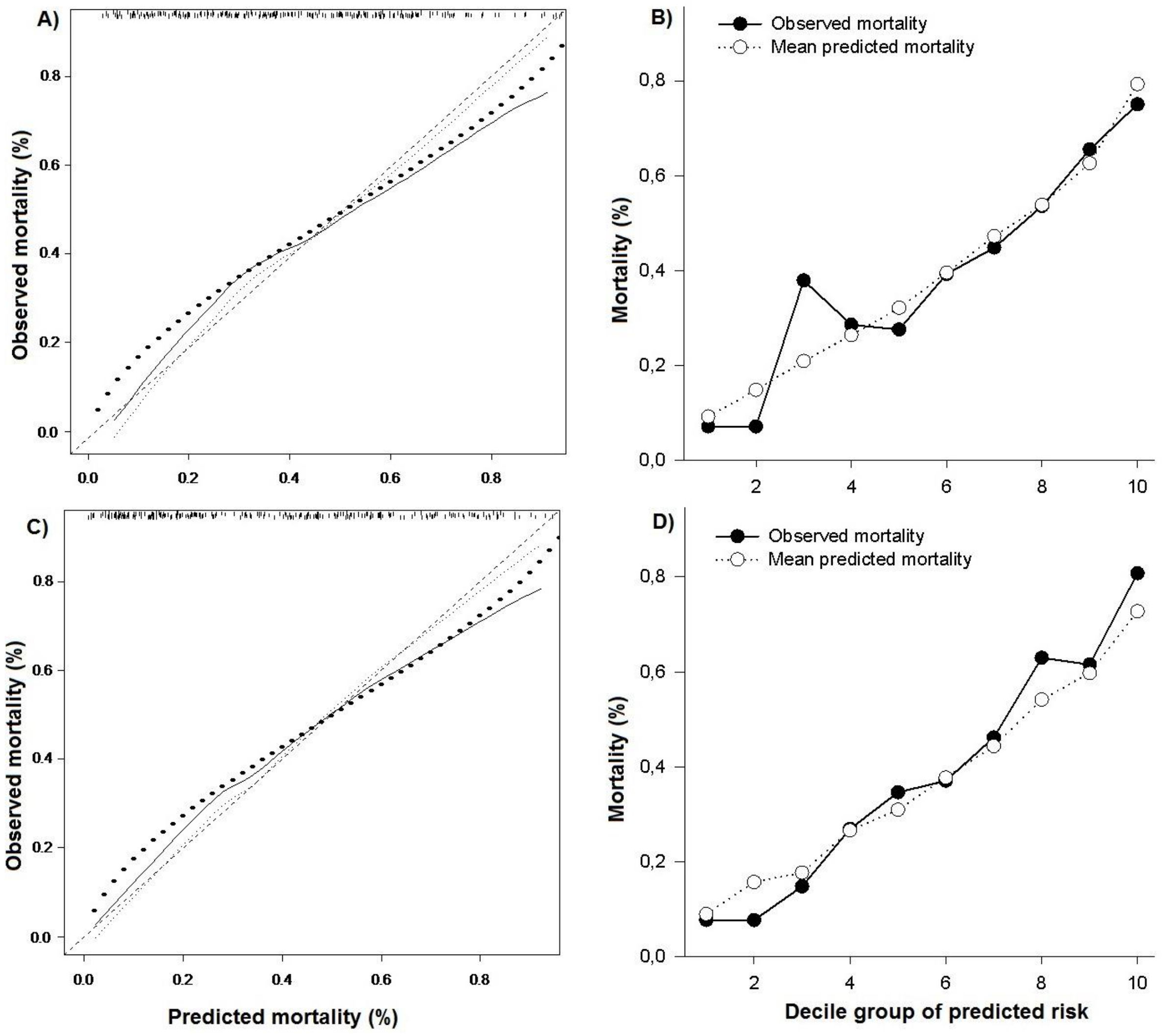

Supplement: Additional file 5 — Observed versus predicted probabilities estimated by Model 1 and Model 2. A figure comparing the relationship between observed and predicted mortalities as well as model calibration for Models 1 and 2. [file cc13824-S5.pdf]
